# Magnetism and magnetoresistance in the critical region of a dilute ferromagnet

**DOI:** 10.1038/s41598-021-81893-2

**Published:** 2021-01-27

**Authors:** M. Wang, B. Howells, R. A. Marshall, J. M. Taylor, K. W. Edmonds, A. W. Rushforth, R. P. Campion, B. L. Gallagher

**Affiliations:** School of Physics and Astronomy, University of Nottingham, University Park, Nottingham, NG7 2RD UK

**Keywords:** Spintronics, Magnetic properties and materials, Ferromagnetism, Phase transitions and critical phenomena

## Abstract

We present detailed experimental measurements and simulations of the field-dependent magnetization and magnetoresistance in the vicinity of the Curie temperature in the highly disordered dilute ferromagnetic semiconductor (Ga,Mn)As. The observed dependence of the magnetization on external magnetic field and temperature is consistent with three-dimensional Heisenberg equation of state calculations including a narrow distribution of critical temperatures. The magnetoresistance shows a peak at the Curie temperature due to the suppression of magnetic scattering in an applied magnetic field, which is well-described by considering changes in the square of the magnetization induced by the magnetic field.

## Introduction

The critical behavior of ferromagnetic materials around their Curie temperature (*T*_C_) is of fundamental interest. Close to *T*_C_, magnetic properties such as magnetization and magnetic susceptibility are determined by critical fluctuations and show power-law behavior^1^. The electrical resistivity and the magnetization are closely related, with short-range magnetization fluctuations giving rise to a temperature-dependence of the resistivity and resulting in a resistive anomaly at *T*_C_^2^. In addition, in ferromagnets the electrical resistivity tensor is dependent on the orientation of the magnetization, giving rise to longitudinal and transverse anisotropic magnetoresistance (AMR) effects^3^.

Dilute ferromagnetic semiconductors, in which a few percent transition metal ions are substituted into a non-magnetic matrix, represent a model strongly disordered system^4^. In particular, (Ga,Mn)As has been widely studied as a testbed for studies of spintronics and magnetoresistance phenomena^5–8^. The Mn dopant is an acceptor when it substitutes for Ga, providing both localized magnetic moments and delocalized holes. The ferromagnetism in (Ga,Mn)As is due to the interaction between the magnetic moments and holes. More recent studies have identified a wide range of dilute ferromagnetic semiconductor systems with independent carrier and spin doping^9,10^.

Studies of critical behavior of the magnetization in dilute ferromagnetic semiconductors have produced disparate and contradictory results^11–15^ (see Ref.^16^ for a detailed discussion). In a previous study^16^, we obtained the critical exponents in the highly disordered dilute ferromagnetic semiconductor (Ga,Mn)As by fitting of Kouvel–Fisher^17^ plots within the region around 2.5% >|*t*|> 0.5%, where *t* = (*T *− *T*_C_)/*T*_C_ is the reduced temperature. The accessible range of *t* is limited by the strong intrinsic disorder as well as large lengthscale inhomogeneity. Here we extend this study by considering also the field-dependence of magnetization and magnetoresistance in the critical region. While the close connection between magnetization and electrical resistance is very well established^18^, critical magnetoresistance has been little-studied in dilute ferromagnetic semiconductors^19^. The increase of magnetic susceptibility around *T*_C_ leads to an increased ordering of magnetic moments and decreased charge carrier scattering which manifests itself as a negative magnetoresistance (*MR*). Thus, the magnetoresistance should behave critically around *T*_C_^20–23^.

## Results

The study was performed on samples cut from a 25 nm (Ga,Mn)As layer with 12% nominal Mn concentration, grown by low temperature molecular beam epitaxy (MBE) on a GaAs(001) substrate^24^. The sample was annealed at 180 °C, resulting in a measured *T*_C_ = (183.5 ± 0.1) K as obtained from a Kouvel–Fisher plot^16^. Due to small differences in the fluxes and substrate temperature between the center and edge of the wafer, there was a small and approximately linear variation of *T*_C_ across the sample^16^. The sample had uniaxial anisotropy with easy axis along the $[1\overline{1}0]$ crystal direction^25^.

### Magnetization and its temperature derivative

Detailed magnetometry measurements around the Curie temperature were performed on a 4-mm-sized sample. The sample was first field cooled to 2 K to form a single domain state with saturation magnetization (*M*_s_) of 65 emu/cm^3^. To compare with the simulation result, the magnetometry data are presented in dimensionless quantities of reduced magnetization *m* = *M*/*M*_*s*_, reduced magnetic field *h* = *μ*_0_*μ*_B_*H*/*k*_B_*T*_C_ and reduced temperature *t*. Here *μ*_0_ is the vacuum permeability, *μ*_B_ is the Bohr magneton, and *k*_B_ is the Boltzmann constant.

**Figure 1:**
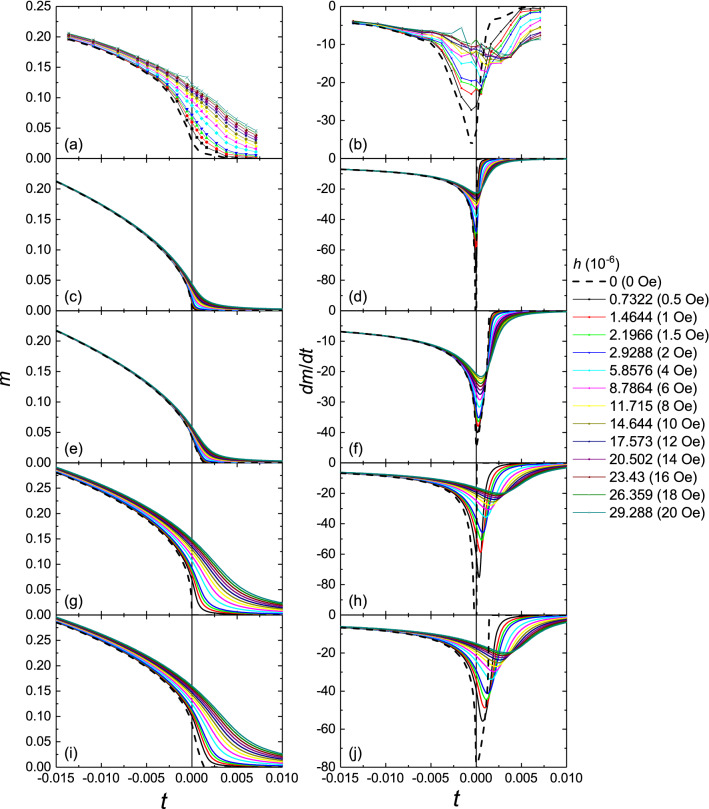
*m* vs. *t* and *dm*/*dt* vs. *t* plots for different applied magnetic fields. (**a**, **b**) Experimental result obtained by SQUID magnetometry for a 25 nm (Ga,Mn)As film. (**c**, **d**) Calculation using the mean field equation of state for a uniform sample. (**e**, **f**) Calculation using the mean field equation of state with 0.5 K *T*_C_ broadening. (**g**, **h**) Calculation using the 3D Heisenberg equation of state for a uniform sample. (**i**, **j**) Calculation using the 3D Heisenberg equation of state with 0.5 K *T*_C_ broadening.

Figure 1a shows the temperature-dependent reduced magnetization (*m* vs. *t*) curves with external magnetic field up to 20 Oe applied along the magnetic easy axis. The magnetization does not drop to zero sharply but has a tail around *T*_C_. The size of the tail is field-dependent and can extend more than 1% *t* (~ 2 K) above the real *T*_C_ when only a few Oe field is applied. Figure 1b shows the temperature derivative of *m* (*dm/dt* vs. *t*) calculated by numerical differentiation. The *dm*/*dt* vs. *t* curve has a sharp minimum around *T*_C_. The peak becomes broader and moves to the right as the magnetic field increases (up to 0.4% of *t*, for *H* = 20 Oe).

Within the critical region, the relation of *m*, *h*, and *t* can be described by an approximate equation of state for the limit of small *t*, *m*, and *h*/*m*^26^:

$${\left(\frac{h}{m}\right)}^{1/\gamma }=at+b{m}^{1/\beta }$$

with model-dependent critical exponents *β*, *γ* and amplitudes *a*, *b*. We first performed the numerical simulation to obtain the *m* vs. *t* curves around *T*_C_ using the mean field model with critical exponents *β* = 0*.5*, *γ* = 1 and amplitudes *a* = 1, *b* = 1/3 for the simulation. We also introduced a rectangular distribution for *T*_C_ of 0.5 K (0.28% or ± 0.14% of *T*_C_) to simulate a sample with a linear variation of *T*_C_ from one side to the other. The calculations (Fig. 1c–f) indicate a much weaker effect of the external magnetic field compared to the experimental result. For a uniform sample, no shift in the minimum of the simulated *dm/dt* is observed as the field increases (Fig. 1d). Even with the inclusion of the 0.5 K *T*_C_ broadening, *the dm/dt* minimum still only shifts by a maximum of 0.05% (Fig. 1f). Hence, the critical behavior of the highly disordered (Ga,Mn)As system is not well described by the mean field equation of state.

Using three-dimensional Heisenberg critical exponents (*β* = 0*.*369, *γ* = 1*.*396) and amplitudes (*a* = 0.82, *b* = 0.4), the simulated *m* vs. *t* curves show similar shape, size and field-dependence of the tail when compared with the experimental data (Fig. 1g–j). The shift of the minimum of *dm/dt* with increasing field is also quantitatively consistent with the experiment. These results indicate that the field- and temperature-dependence of the magnetization in the critical region is well-described by the three-dimensional Heisenberg model.

### Magnetoresistance

**Figure 2:**
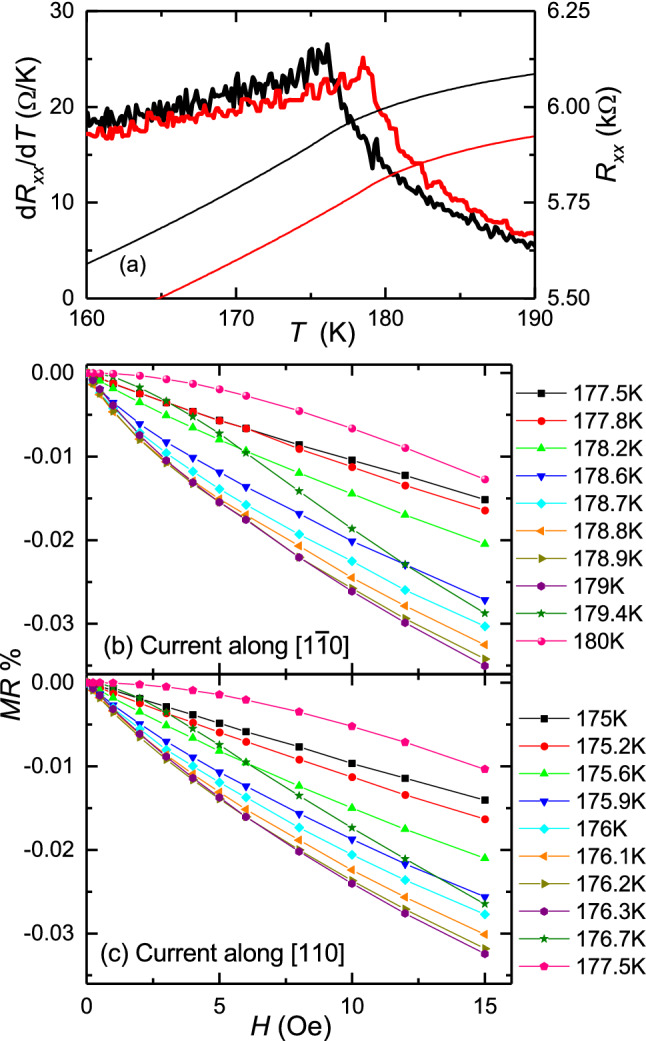
Electrical resistance versus temperature and applied magnetic field around *T*_C_. (**a**) Resistance *R*_*xx*_ (thin lines) and its derivative *dR*_*xx*_*/dT* (thick lines) versus temperature *T*, for Hall bar structures with current along $[1\overline{1}0]$ (red) and [110] (black). (**b**, **c**) Magnetoresistance curves for (**b**) the $[1\overline{1}0]$ and (**c**) the [110] oriented Hall bars, with magnetic field applied along the $[1\overline{1}0]$ axis in both cases.

Magnetoresistance measurements were conducted on two 285 μm × 45 μm Hall bars with current channels along the easy $[1\overline{1}0]$ and hard [110] crystalline axes, respectively. The devices were initially field-cooled from room temperature to 2 K to form a single domain state. Then, the longitudinal resistance (*R*_*xx*_) was measured in zero field as the temperature was increased to above 200 K (Fig. 2a). The Curie temperatures of 178.8 ± 0.2 K and 176.1 ± 0.2 K were estimated from the peak positions in the temperature derivative of the resistivity curves^27^. The reduction of *T*_C_ compared to the magnetometry sample is because the devices were fabricated from different part of the wafer, and possibly also due to the effect of photolithography and etching.

Magnetoresistance measurements were performed at temperatures around *T*_C_ with up to 15 Oe field applied along the $[1\overline{1}0]$ crystal direction (Fig. 2b,c). The magnetoresistance is defined as the change of resistance when applying a magnetic field:

$$MR =\frac{{R}_{\mathrm{xx}}\left(H\right)-{R}_{\mathrm{xx}}\left(0\right)}{{R}_{\mathrm{xx}}\left(0\right)}$$

where *R*_*xx*_(*H*) is the longitudinal resistance in an applied magnetic field of strength *H*. As temperature approaches *T*_C_ from below, the *MR* becomes more and more negative. The *MR* then becomes less negative with a modified shape of the *MR* curve after the temperature rises above *T*_C_. The most negative *MR* curve corresponds to a temperature which is slightly higher than the *T*_C_ obtained from the peak in the temperature derivative of the resistivity. Small differences are observed between the two devices.

We distinguish between anisotropic magnetoresistance (AMR), due to the dependence on the direction of the magnetization relative to the current and crystal, and isotropic MR, which below we relate to the suppression of magnetization fluctuations by the magnetic field. To remove the AMR contribution, we take the average of the |*MR*| for the $[1\overline{1}0]$ and [110] oriented Hall bars at each reduced temperature *t* (temperature normalized using Curie temperatures obtained previously) where the external magnetic field is respectively parallel and perpendicular to the current.

**Figure 3:**
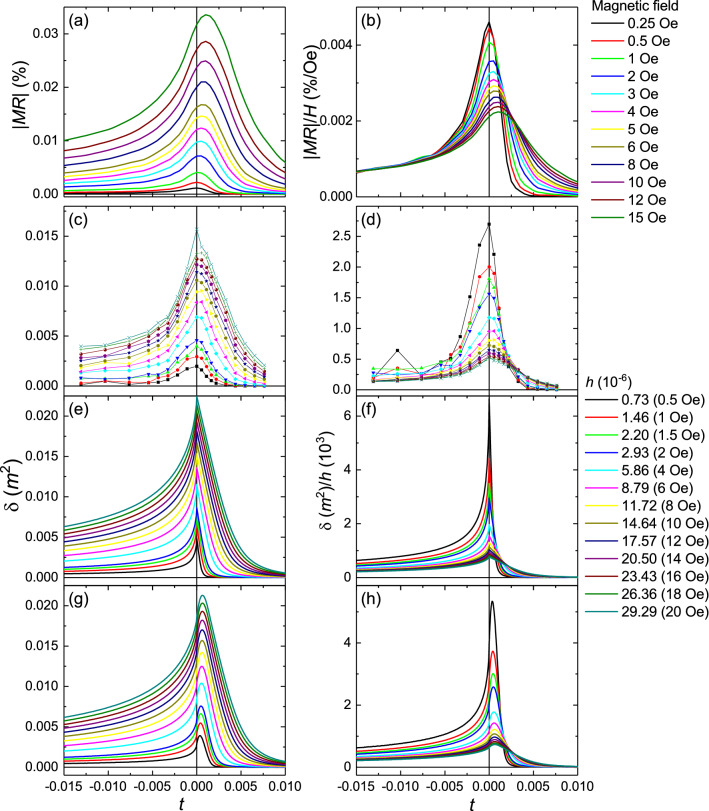
Magnetoresistance and *δ*(*m*^2^) = *m*^2^(*h*) − *m*^2^(0) vs. reduced temperature at different applied magnetic fields. (**a**, **b**) Measured |*MR*| and |*MR*|/*H* vs. *t* for the (Ga,Mn)As Hall bars. Measurements for currents along $[1\overline{1}0]$ and [110] have been averaged to remove the anisotropic magnetoresistance. (**c**, **d**) δ(*m*^2^) vs. *t* and δ(*m*^2^)/*h* vs. *t* calculated from the magnetometry measurements shown in Fig. 1. (**e**, **f**) Numerical simulation of δ(*m*^2^) vs. *t* and δ(*m*^2^)/*h* vs. *t* using the three-dimensional Heisenberg model for a uniform sample. (**g**, **h**) Simulation including *T*_C_ broadening of 0.5 K.

Figure 3a,b show the resulting |*MR*| as well as |*MR*|/*H* versus reduced temperature for different values of *H*. The curves show a clear peak at *t* = 0, which broadens and shifts slightly to higher temperatures with increasing magnetic field. The peak is particularly pronounced for the |*MR*|/*H* curves, with a full width at half maximum of around 0.5% (1 K) for the lowest magnetic fields used.

Magnetization fluctuations in a ferromagnet close to *T*_C_ result in a contribution to the electrical resistivity, which in the lowest order approximation is proportional to [1 − *m*(*H*,*T*)^2^]^18,20–23^. Hence, the relationship between the magnetoresistance and the magnetization in the critical region can be described as:

$$\left|MR\right|\propto \left({m}^{2}\right)$$

where $\left({m}^{2}\right)={m}^{2}\left(h\right)-{m}^{2}\left(0\right)$. Figure 3c,d show the plots of δ(*m*^2^) and δ(*m*^2^)/*h* vs. *t*, obtained from the magnetometry data. Similar to the magnetoresistance behavior, the curves are sharply peaked at *t* = 0, with a crossover of the δ(*m*^2^)/*h* curves at *t* > 0. Simulated curves using the three-dimensional Heisenberg equation of state (Fig. 3e–h) are consistent with both the magnetoresistance and magnetometry experimental results. They also show a clear effect of inhomogeneity, with significant broadening and shift of the peak when a 0.5 K rectangular broadening of *T*_C_ is included.

## Discussion

Our results demonstrate that the magnetoresistance in the critical region of (Ga,Mn)As is well-described by considering only the magnetic field-induced suppression of magnetization fluctuations. The very sharp cusp of *MR/H*, due to its dependence on the square of the magnetization at a given magnetic field, provides a unique probe of the ferromagnetic phase transition. It can be used for accurate determination of *T*_C_, which is an important benchmark property of dilute ferromagnetic semiconductors^5,9,10,19,24,27,28^.

Calculations using an approximate equation of state with three-dimensional Heisenberg-like critical exponents are in good agreement with the measured magnetic field-dependent magnetization in the critical region, and by extension also with the magnetoresistance. In contrast, mean-field critical exponents give a substantial reduction of the field-induced magnetization at temperatures around *T*_C_, compared to the measured data. The mean-field model underestimates the suppression of magnetization fluctuations by an applied magnetic field around *T*_C_^29^. As a result, the mean-field calculated field-dependence of the peak position of *dm/dt* is rather weak.

The observed agreement of field-dependent magnetization of (Ga,Mn)As in the critical region around *T*_C_ is well-described by calculations with the 3D Heisenberg model. This is consistent with our previous study of the thermoremanent magnetization in a similar set of samples, for which 3D Heisenberg-like critical exponents were obtained using Kouvel–Fisher analysis^16^. However, due to the intrinsic disorder present in randomly substituted dilute ferromagnetic semiconductors such as (Ga,Mn)As, the accessible critical region is limited to reduced temperatures |*t*|< 0.025. Any long-range inhomogeneities in the samples will further limit the critical region and may be responsible for some apparently contradictory results in earlier studies^11–15^. Hence, the observed good agreement of the field-induced magnetization with the three-dimensional Heisenberg model, as shown in Fig. 1, provides an important validation of the results of Ref.^16^. This does not rule out the possibility that other universality classes may be found by tailoring the properties of the material, for example by inducing a strong uniaxial magnetic anisotropy by growth on relaxed (In,Ga)As buffer^30^. The dependence of *T*_C_ (x) in (Ga_1-x_,Mn_x_)As (shown in e.g. Ref.^24^) points to a long-range character of the spin–spin interactions.

The magnetization and magnetoresistance measurements and calculations further demonstrate the marked effect of even a moderate broadening of ∆*T*_C_ = 0.5 K =  ± 0.14%. The peak in the calculated δ(*m*^2^)*/h* becomes broader and shifts to higher temperature when the broadening is included. The effect of broadening due to inhomogeneity can also be observed in the experimental magnetoresistance traces; for example, the largest |*MR*| is observed at a higher temperature than the *T*_C_ estimated from the *dR*_*xx*_/*dT* curves.

## Methods

### Sample preparation

A 25 nm of (Ga,Mn)As with 12% nominal Mn concentration was grown on a 2 in. GaAs(001) wafer at 200 °C by molecular beam epitaxy. The sample was annealed at 180 °C for 48 h to remove the interstitial Mn. Hall bars with current channel width of 45 μm, and voltage probes separated by 285 μm, were fabricated by standard photolithography with 20 nm Ti/100 nm Au contact pads for four terminal transport measurements.

### Magnetometry measurement

The 4-mm-sized sample was measured using a Quantum design MPMS system. The sample first has been measured at 2 K to obtain the saturation magnetization. Then the system has been carefully demagnetized to reduce the remanent field within magnet coil to less than 0.5 Oe, before performing the detailed measurements around Curie temperature.

### Magnetotransport measurement

4 terminal DC transport measurements were performed in an Oxford Instruments cryostat system. A Keithley 2400 Sourcemeter was used for applying 0.1 mA constant current along the Hall bars and a Keithley 2000 Multimeter was used for measuring the longitudinal voltages. The cryostat sample space and electromagnetic coil were covered by a mu-metal cylinder to provide magnetic shielding. The remanent field within the sample space is less than 0.1 Oe measured by a calibrated Hall probe.

### Numerical simulation

The numerical simulation was coded in Python language. Newton–Raphson method was used to find the magnetization values giving roots of the equations of state at each temperature and magnetic field.
